# Genome Sequence of *Pseudomonas* sp. HUK17, Isolated from Hexachlorocyclohexane-Contaminated Soil

**DOI:** 10.1128/genomeA.00275-16

**Published:** 2016-04-14

**Authors:** Cyrielle Gasc, Jean-Yves Richard, Pierre Peyret

**Affiliations:** a EA 4678 CIDAM, Université d’Auvergne, Clermont-Ferrand, France; b SITA Remediation, Suez, Meyzieu, France

## Abstract

*Pseudomonas* sp. HUK17 has been isolated from hexachlorocyclohexane (HCH) long-term contaminated soil. The genome of strain HUK17 was sequenced to elucidate its adaptation toward HCH and to evaluate the presence of pesticide degradation pathways. Here, we report the annotated draft genome sequence (~2.6 Mbp) of this strain.

## GENOME ANNOUNCEMENT

For years, hexachlorocyclohexane (HCH) has been widely used around the world to control agricultural pests because of its insecticidal properties (1). Today, many countries have restricted or prohibited its use. Indeed, HCH is highly toxic and persistent in the environment (2). Bioremediation strategies can be relevant and promising to clean up contaminated agricultural and industrial sites, and these approaches require a thorough study of metabolic capabilities of indigenous soil microbial communities (3). In this context, an aerobic HCH-supplemented liquid cultivation of microorganisms from soil contaminated with HCH in an ancient chemical factory (Huningue, France) has been carried out, and subsequent repeated seeding of individual colonies has led to the isolation of the *Pseudomonas* sp. HUK17 (SITA Remediation, Suez). Further study of this strain demonstrated its HCH degradation capabilities, given its growth in minimal salt media containing HCH as the sole carbon.

Thus, to better understand HCH genetic adaptation and potential degradation mechanisms of strain HUK17, its genomic DNA was sequenced by use of an Illumina HiSeq 2500 platform (CASAVA version 1.8.2). The shotgun sequencing generated 2,247,813 high-quality paired-end reads. Reads were first quality-trimmed using Trimmomatic version 0.32 (4). They were then assembled *de novo* with Velvet version 1.2.10 (5) and annotated using the NCBI Prokaryotic Genome Annotation Pipeline (PGAP) (http://www.ncbi.nlm.nih.gov/genome/annotation_prok). The draft genome consists of 15 contigs totaling 2,568,003 bp, with a G+C content of 65.43%. Contigs have an *N*_50_ length of 236 kb and an average length of 171.20 kb, the largest contig being 455.65 kb. Gene prediction and annotation identified 2,272 coding sequences and 24 pseudogenes.

Currently characterized HCH-degrading microbial species require the *linA* to *linJ* genes coding for enzymes involved in different convergent HCH degradation pathways (6). Consequently, a BLAST analysis was performed to search for corresponding gene sequences of *Sphingobium indicum* B90A (accession no. AJXQ00000000) within the strain HUK17 genome. None of these genes or their homologues were identified, and no other gene likely to be involved in HCH degradation was evidenced. Thus, in accordance with cultural observations, these results could indicate that *Pseudomonas* sp. HUK17 degrades HCH using other currently undescribed metabolic pathways, as has been suggested in other studies (6–8).

### Nucleotide sequence accession numbers.

This whole-genome shotgun project has been deposited in DDBJ/ENA/GenBank under the accession number LSMW00000000. The version described in this paper is the first version, LSMW00000000.1.
